# Aphasia and herpes virus encephalitis: a case study

**DOI:** 10.1590/S1516-31802012000500011

**Published:** 2012-11-13

**Authors:** Ellen Cristina Siqueira Soares-Ishigaki, Maysa Luchesi Cera, Alexandre Pieri, Karin Zazo Ortiz

**Affiliations:** I Doctoral Student in Human Communication Disorders at Universidade Federal de São Paulo (Unifesp), São Paulo, Brazil.; II Speech Therapist. Master’s Student in Human Communication Disorders at Universidade Federal de São Paulo (Unifesp), São Paulo, Brazil.; III MD. Doctoral Student in Neurology at Universidade Federal de São Paulo (Unifesp), São Paulo, Brazil.; IV PhD. Speech Therapist and Professor of the Department of Speech Therapy, Universidade Federal de São Paulo (Unifesp), São Paulo, Brazil.

**Keywords:** Meningoencephalitis, Encephalitis, herpes simplex, Aphasia, Language, Cognition, Adolescent, Meningoencefalite, Encefalite por herpes simples, Afasia, Linguagem, Cognição, Adolescente

## Abstract

**CONTEXT:**

Meningoencephalitis early in life, of any etiology, is a risk factor for development of subsequent sequelae, which may be of physical, psychiatric, behavioral or cognitive origin. Anomia is a language abnormality frequently found in such cases, and other language deficits are rarely described. The aim of this study was to describe the cognitive and linguistic manifestations following a case of herpetic meningoencephalitis in a 13-year-old patient with eight years of schooling.

**CASE REPORT:**

The patient underwent a speech-language audiology assessment nine months after the neurological diagnosis. The battery of tests included the Montreal-Toulouse Language Assessment test protocol (MT Beta-86, modified), the description from the Cookie Theft task of the Boston Diagnostic Aphasia Examination (BDAE), an informal assessment of the patient’s logical and mathematical reasoning, and the neuropsychological subtests from the WAIS-III scale, which assess working memory. The patient presented mixed aphasia, impairment of short-term memory and working memory, and dyscalculia. This case also presented severe cognitive and linguistic deficits. Prompt diagnosis is crucial, in order to enable timely treatment and rehabilitation of this neurological infection and minimize the cognitive deficits caused by the disease.

## RESUMO

**CONTEXTO:**

Meningoencefalite em idade precoce, de qualquer etiologia, é fator de risco para o desenvolvimento de sequelas posteriores, que podem ser de origem física, psíquica, comportamental ou cognitiva. Anomia é uma manifestação linguística frequente nesses casos e outras alterações de linguagem raramente são descritas. O objetivo do estudo foi descrever as manifestações linguísticas e cognitivas pós-meningoencefalite herpética de um paciente com 13 anos de idade e oito anos de escolaridade.

**RELATO DE CASO:**

O paciente passou por avaliação fonoaudiológica nove meses após o diagnóstico neurológico. A bateria de avaliação incluiu o Protocolo de Avaliação de Linguagem Montreal Toulouse (MT Beta-86 modificado), a descrição da prancha Roubo dos Biscoitos do Teste de Boston para o diagnóstico da afasia, avaliação informal do raciocínio lógico-matemático e os subtestes neuropsicológicos da escala WAIS-III que avaliam a memória de trabalho. O paciente apresentou afasia mista, alteração da memória de curto prazo e da memória de trabalho e discalculia. Apresentou também alterações cognitivas e linguísticas graves. O diagnóstico precoce tem fundamental importância para o tratamento oportuno e a reabilitação dessa infecção neurológica e também para minimizar as alterações cognitivas causadas pela doença.

## INTRODUCTION

Herpesvirus infection can cause acute viral encephalitis and various neurological disorders, including meningoencephalitis. Herpes simplex virus encephalitis is a serious disease, with high levels of morbidity and mortality.^1^ Studies have shown that meningoencephalitis early in life is a risk factor for development of psychiatric, behavioral or cognitive complications later in life^2^^,^^3^^,^^4^^,^^5^^,^^6^^,^^7^^,^^8^ and have revealed the need for prompt diagnosis of the condition. The complexity of the differential diagnoses for meningoencephalitis hampers diagnosis of the exact etiology in these cases.^9^ Diagnostic delay is associated with poorer prognosis, because it delays the commencement of treatment, thus leading to deterioration in the patient’s overall status.^9^

Okuda et al. reported on a patient with pure anomic aphasia following encephalitis, in which the patient’s naming difficulty persisted without other dysfunction of language or memory.^3^ Other studies have reported on patients who developed herpes simplex encephalitis involving the left temporal lobe, with resultant aphasia.^3^^,^^4^^,^^5^ Sudden onset of language abnormalities (aphasia) is therefore an important sign suggestive of herpes simplex encephalitis.

Nonetheless, some neuropsychological studies have reported that anomia is one of the most frequently found language abnormalities in cases of post-herpetic meningoencephalitis.^2^^,^^3^^,^^4^^,^^5^^,^^6^^,^^7^^,^^8^ Other language deficits have only rarely been described in these individuals.^5^ Early diagnosis makes it possible to establish better criteria for optimal rehabilitation of linguistic and cognitive difficulties in this patient group. Thus, we hypothesized that after a case of herpetic meningoencephalitis, individuals would present language and cognitive deficits.

The aim of this study was to describe a case subsequent to herpetic meningoencephalitis in which the linguistic and cognitive disorders were protean.

## CASE REPORT

The case subject was a right-handed male aged 13 years and 3 months with 8 years of schooling. He was a Portuguese-speaking native of the city of São Paulo. He had not had any previous language problems or learning difficulties and had never repeated a school year.

His parents gave informed consent for his case to be reported, and the study was approved by our institution’s Research Ethics Committee (CEP no. 0151/05).

In December 2005, the boy’s parents took him to the Emergency Department at one of the city’s private hospitals because he presented with confusion, disconnected speech, migraine, fever and nuchal rigidity. Five days after onset of symptoms, the patient was admitted to the same hospital. A diagnosis of herpes meningoencephalitis was reached based on cerebrospinal fluid (CSF) examination. A hemogram and computed tomography scan were performed on the day after admission and did not reveal any abnormalities.

After 21 days in hospital, the patient’s condition had improved and he was discharged. At this point, he was able to recognize his home and his bedroom. However, he was readmitted three days later due to a recurrence of herpetic meningoencephalitis. At this stage, he did not recognize his friends or family, nor was he able to understand facts or phrases. He was discharged after a further 10 days, and was able to recognize his family members and speak using simple words and phrases. One month later, he went back to school but did not recognize or interact with his friends.

In September 2006, he underwent a speech-language audiology evaluation at the Speech Therapy and Neurolinguistic Investigation Unit of the Department of Speech Therapy, Universidade Federal de São Paulo - Escola Paulista de Medicina (Unifesp-EPM), because his parents noted that he still had difficulty in understanding and speaking, and forgot names.

The Montreal-Toulouse Language Assessment test (MT Beta-86, modified)^10^ was applied. This consists of tests to characterize oral and written production and comprehension, as well as repetition and fluency. The means obtained from the test were compared with the means for normal controls published by Soares and Ortiz.^11^ Oral production was tested using the Cookie Theft task of the Boston Diagnostic Aphasia Examination (BDAE).^12^ The patient’s logical and mathematical reasoning abilities were also assessed based on oral and written calculations using the four mathematical operators. The Corsi Block^13^ and Digit Span items of the WAIS-III scale were applied to evaluate working memory in forward and reverse order.^14^ To complete the examination, the patient was asked to categorize everyday objects, and a word dictation task was administered to evaluate writing abilities.

**Figure 1.**
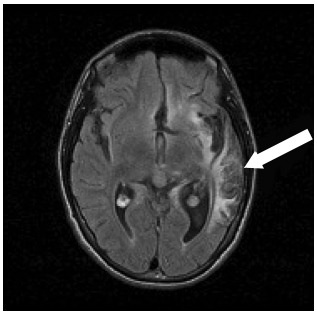
Magnetic resonance imaging performed in May 2007 showing areas of encephalomalacia with principal involvement of the left temporal lobe, and lesser involvement of the left frontal lobe. These represent areas typically affected by herpes simplex virus.

**Figure 2.**
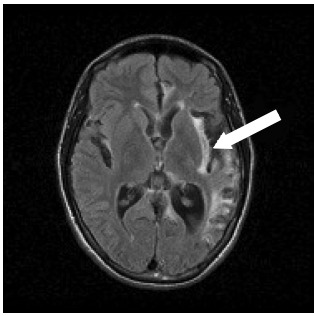
Magnetic resonance imaging showing areas of encephalomalacia with principal involvement of the left temporal lobe, and lesser involvement of the left frontal lobe. These represent areas typically affected by herpes simplex virus.

In May 2007, the patient underwent a magnetic resonance imaging (MRI) examination, which showed areas of encephalomalacia predominantly involving the left temporal lobe, and to a lesser extent, the left frontal lobe, right temporal lobe, precuneus and lateral region of the left occipital lobe. The images from the MRI examinations performed in May 2007 are shown in Figures 1 and 2.

For better understanding of the patient’s current status, and thus to enable better disease management and treatment planning, the cognitive and language manifestations were correlated with models that explain oral and written linguistic processes.^15^^,^^16^^,^^17^

The results from the cognitive and linguistic evaluations, along with the respective tests used, are shown in Table 1.

**Table 1.** Patient’s performance in tasks from the MT Beta-86 (modified) language assessment test, including expected scores based on his level of schooling, according to Soares and Ortiz^11^

Tasks from MT Beta 86 (modified)	Score obtained	Expected score
Interview	5.0	11.8
Automatisms - form	10.0	29.0
Automatisms - content	1.0	3.0
Oral comprehension	27.0	39.3
Repetition	18.0	32.2
Reading	6.0	32.4
Written comprehension	3.0	12.5
Naming	2.0	28.1
Verbal fluency	0.0	18.7
Orofacial praxis	4.0	5.8
Oral comprehension (body parts)	0.0	7.9
Object manipulation	2.0	8.0
Copying	4.0	4.0
Dictation	0.0	11.8
Written comprehension (body parts)	0.0	7.9
Written naming (actions)	1.0	5.8
Number repetition	3.0	10.0
Number reading	3.0	9.9

The data in Table 1 reveal that the patient achieved lower-than-expected scores (for the Brazilian population)^11^ in all tasks of the Montreal-Toulouse Protocol, for his level of schooling.

The patient presented moderate deficit of oral comprehension, and his speech showed phonemic and semantic paraphasia, frequently forgetting of words, circumlocutions and perseverations. In reading aloud tasks, he presented verbal, phonological and formal paralexia, and perseveration. The patient had serious difficulties in understanding writing. During his written production, he produced literal paragraphs and graphemes. In the word dictation assessment using words identified orally, the pattern of errors remained unchanged.

In addition to the signs described, the patient showed problems on a pragmatic level: searching for words, vague expression of ideas and repeated production of scenes, as shown in the Cookie Theft task of the BDAE. These pragmatic problems are not evaluated in functional neurocognitive language models, given that these models evaluate word processing.

In our case, although anomia was also the most important deficit, other important cognitive and linguistic signs were also evident. Table 2 shows the results from reviewing the medical databases using descriptors for the main clinical findings observed in our patient. Several studies have reported cases with anomia subsequent to meningoencephalitis caused by herpes simplex.^2^^,^^3^^,^^4^^,^^5^^,^^6^^,^^7^^,^^8^ Few studies have characterized the language deficits.^5^^,^^18^ Lowe, Knapp and Lambon Ralph reported that jargon was used and there was phonological impairment, anomia and mild impairment of comprehension.^5^ In our study, the language difficulties and other cognitive functions relating to language were thoroughly characterized in a patient who was a speaker of Brazilian Portuguese. The patient in our study started to attend a speech therapy clinic only after nine months had elapsed following his brain injury, and did so because it was noticed that he presented significant difficulty with regard to naming, reading, writing and oral and written comprehension.

**Table 2.** Results from our review of the medical databases, performed on May 13, 2011, using descriptors for the main clinical findings observed in our patient

Search strategy	Database	Results
“anomia” AND “herpes simplex” AND (“last 10 years”[PDat])	PubMed	3
	Lilacs	0
	SciELO	0
	Embase	6
“language” AND “herpes simplex” AND “cognition” AND (Meningoencephalitis OR Encephalitis) AND (“last 10 years”[PDat])	PubMed	6
	Lilacs	0
	SciELO	0
	Embase	52
“aphasia” AND “herpes simplex” AND “cognition” AND (“last 10 years”[PDat])	PubMed	2
	Lilacs	0
	SciELO	0
	Embase	15

By applying the abovementioned models for explaining oral and written linguistic processes,^15^^,^^16^^,^^17^ we were able to determine that our patient had an intact auditory perception analysis system, since he was able to differentiate nonverbal sounds from verbal sounds. In the oral comprehension task on high-frequency words in Brazilian Portuguese, one of the two errors made had no semantic correlation with the stimulus presented (he pointed to a spade instead of a knife). Also, at other points during the evaluation, the patient was unable to ascertain whether a word uttered by the examiner belonged to the Portuguese language. The observed deficits may have been due to a compromised phonological input lexicon, semantic lexicon dissociation and/or problems with the lexical buffer or in accessing these storage areas. Given that the patient performed better in tasks involving repetition, we believe that the deficit was unlikely to have been caused by alteration in the phonological input lexicon, but rather, was due to semantic lexicon dissociation and/or a lexical buffer problem.

The other error was semantically correlated with the stimulus solicited, since the patient pointed to a drawing showing “hair” when the correct response was “comb”. He was unable to identify all eight body parts, although he did correctly identify the semantic fields that corresponded to the stimuli. These deficits may have been related to a failure in accessing the semantic lexicon.

With regard to naming tasks, the patient was able to name only one of the 31 drawings presented. The problems manifested during this task consisted of four paraphrases and 26 instances of anomia. The paraphrasing indicated that he was able to visually recognize the stimuli and access the semantic system but, despite this, the abnormality may have resulted from failure in accessing the lexical buffer. The deficits underlying the anomia could have occurred in any phase of linguistic processing, since the ability to name, in tasks requiring names for drawings, involves analysis and recognition of visual elements (lines, bars, points and curves), in order to form a complex visual representation of an object. Through the subject’s internal recognition process and experiences, the image generates a mental representation. Subsequent to this process, the object gains representation in the semantic system and correct phonological lexicon activation finally takes place.^19^

With regard to repetition tasks, our patient had greatest difficulty in repeating real words, as opposed to pseudo-words or non-words. This indicates that the lexical buffer was more compromised than the phonological buffer was. Thus, the patient’s oral production suggested that his phonological input lexicon (also known as the auditory input lexicon) was compromised, along with the semantic and lexical buffers. Biedermann and Nickels assumed that the primary cause of poor word retrieval among patients who had had herpetic encephalitis was their impaired access to phonological representations at the word formation level. This could be due to a combination of post-lexical mapping impairment and mild semantic impairment.^18^

In reading-aloud tasks, we observed that the patient had difficulty with most words, and was able to read only a few closed-class words and some pseudo-words. He managed to read only six out of 33 words: three closed-class words (mine, if and that), one pseudo-word (vica) and two real familiar words (stage and life). The difficulties encountered by the patient in relation to real familiar words, as well as in pseudo-words and unfamiliar words, demonstrated that the deficits could lie in any processing stage and that both pathways were affected. In view of these findings, we concluded that the patient decoded or made greater use of the perilexical pathway. This suggests that when access to semantic knowledge was not required, the patient’s performance improved. It thus shows that the phonological route seemed to be less affected in reading than the lexical route was.

With regard to written production, the patient did not manage to write all the words. In a dictation test on frequent, infrequent, regular and irregular words, the subject presented literal paragraphia and graphemes. These errors made by the patient showed that both writing routes were compromised. Thus, the patient presented deficits independent of the route used or the type of error. In order to eliminate interference from oral comprehension in dictation tasks, a supplementary evaluation was performed based on the words that were adequately understood by the patient in the oral comprehension subtest of the protocol. In this, the pattern of errors remained unchanged, thus corroborating the abnormalities in both the lexical and the perilexical routes. The patient was unable to complete the copying task, which confirmed that regardless of input (auditory or visual), he indeed had a severe writing deficit.

In the written word comprehension task, the patient understood two of the five familiar stimuli. This abnormality of written comprehension could have stemmed from a breakdown at any stage of processing, from the visual input to the semantic processing.

Using the language processing models, we found that the patient had deficits in both the lexical and the perilexical routes. However, it was noted that his performance in the oral and written tasks, especially in the repetition test, suggested that the sublexical processes were less affected. Semantic impairment in presentations of herpes simplex virus encephalitis has been described in previous studies.^4^^,^^5^^,^^6^^,^^7^^,^^8^ The study by Biedermann and Nickels showed that the level at which word production breaks down can be localized between the semantic and the phonological processes.^18^

In the Corsi Block test, the patient correctly identified seven forward sequences and six backward sequences. In the Digit Span test, he correctly performed two algorithms forwards but was unable to perform them in reverse order.

The patient was able to correctly add and subtract up to three digits, but did not recognize multiplication and division signs or know how to perform these arithmetical operations.

With regard to categorization, personal hygiene items (soap, shampoo and toothpaste), kitchen utensils (spoon, fork, knife and cup), musical instruments (bell and recorder), games (dominoes, videogame remote control and videogame cartridge) and school materials (pencil case, pencil, retractable pencil, eraser, ballpoint pen and felt-tip pen) were presented simultaneously. However, after instructions were provided for the task, he showed no interest in performing it. Other simple categorization tasks were attempted, but the patient proved unable to accomplish them. Unfortunately, it was not possible to delve deeper into the linguistic and cognitive deficits of our young patient because his severe oral comprehension difficulties hampered his understanding of instructions given.

The Corsi Block and Digit Span neuropsychological tests were used to assess the cognitive functions of attention, working memory and short-term memory, and executive function. These tests contain stimuli that activated both the visual and the auditory input routes. Upon auditory presentation of the stimulus, the patient’s performance was poorer than would be expected according to Figueiredo and Nascimento.^20^ However, he performed well in the Corsi Block test. This indicated that tests involving auditory information processing tasks were more difficult to perform than visual tests were, which was consistent with the location of his cerebral lesions. The imaging examination showed that the majority of the left temporal lobe was compromised. Likewise, but to a lesser extent, the left frontal lobe, right temporal lobe, precuneus and lateral aspect of the left occipital lobe were also compromised. According to Noppeney and Price, the temporal and frontal lobes interact in oral comprehension such that voice sounds are analyzed in the temporal lobe while the frontal lobe analyzes syntax and theme.^21^ The message then undergoes semantic lexicon and semantic syntax processing, after which the subject can understand the oral information. The lesions shown on magnetic resonance imaging in our patient affected the regions that play a role in processing information for oral comprehension, which in turn involve processing of auditory information. The patient’s performance impairments in the auditory tasks outlined above are congruent with these imaging findings. In addition to the fact that the compromised performance in the Digit Span test were consistent with the abnormalities in language tasks described above, the results from this test showed that there were deficits of attention, working memory and short-term memory in processing the auditory information.

Manifestations similar to those seen in our patient were also described by Pewter et al., who reported alterations in executive functions, language and working memory in patients with encephalitis.^2^ In addition to these problems, our patient also had difficulty in performing mathematical calculations.

In mathematical tests, the patient could not remember the rules of arithmetic. Although he recognized the numbers, he was unable to identify the signs for multiplication and division, and these symptoms point towards a diagnosis of acalculia. Similarly, Souza also observed slowing down of information processing and difficulties in mathematical calculations, in a study involving an adult female patient with herpetic meningoencephalitis.^22^ Our patient also presented difficulty in performing calculations and in cognitive factors, but more specifically in relation to working memory, while also showing signs of executive function impairment. Cognitive deficits may interfere with calculation abilities, and therefore, in addition to the patient’s difficulties in recognizing the multiplication and division signs, his cognitive deficits may also have interfered with his test performance. De Luccia et al. showed that multiplication is processed predominantly through the phonological loop, while subtraction is mediated principally by the visuospatial sketchpad.^23^ The patient had greater deficits in multiplication than in subtraction, and this finding is congruent with worse compromising of the phonological loop than of the visuospatial sketchpad. This is also consistent with the poorer performance in the auditory task (Digital Span test) than in the visual task (Corsi Block test).

Our patient not only presented abnormalities in calculation processing and lexical access, but also showed difficulties in working memory. Similarly, other studies have confirmed that aphasic individuals show deficits in working memory and numerical processing.^24^^,^^25^ The hypothesis put forward is that calculation processing difficulties in aphasic individuals could be related to oral and written comprehension deficits, which would represent abnormalities in lexical and visuospatial access.^25^^,^^26^

## CONCLUSION

In summary, we were able to document serious compromising of cognitive functions such as language, memory and ability to perform mathematical tasks, in this patient with herpetic meningoencephalitis. This case study involving a young patient highlights the importance of reaching an early diagnosis in order to adequately treat the neuroinfection, decrease the severity of the sequelae from encephalitis and reduce the impact of cognitive deficits.
